# Development of a clinical prediction rule to diagnose *Pneumocystis jirovecii* pneumonia in the World Health Organization’s algorithm for seriously ill HIV-infected patients

**DOI:** 10.4102/sajhivmed.v19i1.851

**Published:** 2018-07-23

**Authors:** Gary Maartens, Annemie Stewart, Rulan Griesel, Andre P. Kengne, Felix Dube, Mark Nicol, Molebogeng X. Rangaka, Marc Mendelson

**Affiliations:** 1 Division of Clinical Pharmacology, Department of Medicine, University of Cape Town, South Africa; 2 Non-Communicable Diseases Research Unit, South African Medical Research Council, South Africa; 3 Division of Medical Microbiology, Department of Pathology, University of Cape Town, South Africa; 4 National Health Laboratory Service, South Africa; 5 Department of Medicine and School of Public Health, University of Cape Town, South Africa; 6 Institute for Global Health, Department of Infection and Population Health, University College London, United Kingdom; 7 IIDMM, University of Cape Town, South Africa; 8 Division of Infectious Diseases and HIV Medicine, Department of Medicine, University of Cape Town, South Africa

## Abstract

**Background:**

The World Health Organization (WHO) algorithm for the diagnosis of tuberculosis in seriously ill HIV-infected patients recommends that treatment for *Pneumocystis jirovecii* pneumonia (PJP) should be considered without giving clear guidance on selecting patients for empiric PJP therapy. PJP is a common cause of hospitalisation in HIV-infected patients in resource-poor settings where diagnostic facilities are limited.

**Methods:**

We developed clinical prediction rules for PJP in a prospective cohort of HIV-infected inpatients with WHO danger signs and cough of any duration. The reference standard for PJP was > 1000 copies/mL of *P. jirovecii* DNA on real-time sputum polymerase chain reaction (PCR). Four potentially predictive variables were selected for regression models: dyspnoea, chest X-ray, haemoglobin and oxygen saturation. Respiratory rate was explored as a replacement for oxygen saturation as pulse oximetry is not always available in resource-poor settings.

**Results:**

We enrolled 500 participants. After imputation for missing values, there were 56 PJP outcome events. Dyspnoea was not independently associated with PJP. Oxygen saturation and respiratory rate were inversely correlated. Two clinical prediction rules were developed: chest X-ray possible/likely PJP, haemoglobin ≥ 9 g/dL and either oxygen saturation < 94% or respiratory rate. The area under the receiver operating characteristic curve of the clinical prediction rule models was 0.761 (95% CI 0.683–0.840) for the respiratory rate model and 0.797 (95% CI 0.725–0.868) for the oxygen saturation model. Both models had zero probability for PJP for scores of zero, and positive likelihood ratios exceeded 10 for high scores.

**Conclusion:**

We developed simple clinical prediction rules for PJP, which, if externally validated, could assist decision-making in the WHO seriously ill algorithm.

## Introduction

*Pneumocystis jirovecii* pneumonia (PJP) is a common opportunistic infection in HIV-infected patients. There is a widespread belief that PJP is uncommon in HIV-infected adults in sub-Saharan Africa, but a systematic review reported a prevalence of PJP of 22.4% in inpatients, with a case fatality of 18.8%.^1^ Early recognition and treatment of PJP is important to reduce mortality. The diagnosis of PJP is difficult in low-middle-income countries (LMICs) because facilities for bronchoscopy, sophisticated imaging and microbiologic identification of *P. jirovecii* are limited and because co-infections are common, especially with tuberculosis.^1,2^

The World Health Organization (WHO) algorithm for the diagnosis of tuberculosis in seriously ill HIV-infected patients (defined as current cough, fever, night sweats or weight loss plus presence of one or more of the following danger signs: respiratory rate > 30/min, heart rate > 120/min, temperature > 39 ^°^C and being unable to walk unaided) recommends that treatment for PJP should be considered without giving clear guidance on which seriously ill patients should be selected for empiric PJP therapy.^3^ A recent systematic review of studies conducted in LMICs found that the predictive value of symptoms for the diagnosis of PJP was poor, but chest radiography was of value.^2^ There is thus a need for diagnostic tools for PJP that can be implemented in LMICs.

We developed a clinical prediction rule for the diagnosis of PJP in a prospective cohort study of HIV-infected inpatients meeting WHO criteria for seriously ill using simple clinical and laboratory variables that are readily available in resource-poor settings.

## Methods

We conducted a prospective cohort study in two regional hospitals in Cape Town, South Africa, serving high burden HIV and tuberculosis communities. We have recently reported the development of clinical prediction rules for the diagnosis of tuberculosis from the same cohort.^4^ Inclusion criteria were adults (≥ 18 years), HIV-infected, screened within 24 hours of admission, current cough of any duration and ≥ 1 WHO danger signs. Exclusion criteria were adults on anti-tuberculosis therapy, completed anti-tuberculosis therapy in the previous month, defaulted anti-tuberculosis therapy within the past six months, exacerbation of congestive cardiac failure or chronic obstructive pulmonary disease, and being unable to produce a spontaneous or induced sputum sample.

Demographic and clinical data were recorded on standardised case record forms. In keeping with WHO recommendations, all participants were commenced on empiric broad spectrum β-lactam antibiotics (typically ceftriaxone or amoxicillin-clavulanate), either at the referring clinic or on admission to the hospital emergency unit. On admission, venepuncture was performed for haemoglobin, white cell count (WCC), CD4+ count (unless CD4+ count was performed within the previous six months) and mycobacterial blood culture (BacT/Alert MP; bioMerieux, Durham, NC, USA). Serum (1→3)- β-d-glucan (FungitellÒ; Associates of Cape Cod, Inc., East Falmouth, MA, USA) was obtained after the first 107 participants were enrolled when additional funding became available. Chest radiographs were retrospectively reviewed by a single radiologist (blinded to the diagnosis), who documented specific radiographic features and classified radiographs as likely, possible or unlikely for pulmonary tuberculosis and/or bacterial pneumonia and/or PJP. Pulse oximetry was performed at admission – participants who were able to exercise were exercised to target heart rate and oximetry repeated and the lowest oxygen saturation was recorded.

Sputum induction using an ultrasonic nebuliser and hypertonic saline was performed in participants unable to spontaneously produce sputum. One sputum sample was sent for Gram stain, culture and sensitivity; and for quantitative, multiplex, real-time polymerase chain reaction (PCR) with FTDResp33 (Fast-Track Diagnostics, Esch-sur-Alzet, Luxembourg) to identify potential respiratory pathogens, including *P. jirovecii* (for details of sample preparation, see Dube et al.).^5^ A standard curve was derived using a plasmid standard supplied by the manufacturer, and genome copy number of *P. jirovecii* extrapolated from this curve. The limit of quantification ranged from 1.12 × 10^3^ to 1.6 × 10^8^ copies per reaction. Because of a laboratory error, 43% of the stored sputum samples for PCR were destroyed. Two sputum samples were sent for smear microscopy with auramine staining and liquid mycobacterial culture (BACTEC^TM^ MGIT^TM^ 960; Becton, Dickinson and Company, NJ, USA), and on one of the samples, the Xpert MTB/RIF assay (Xpert MTB/RIF; Cepheid, Sunnyvale, CA, USA). Where appropriate, other specimens (e.g. pleural fluid) were sent for liquid mycobacterial culture.

The research medical officers made a discharge diagnosis based on clinical and radiographic features, available microbiological data and response to therapy – more than one diagnosis could be made. Results of serum β-d-glucan and sputum real-time PCR were not available to the medical officers.

### Statistical analysis

Analyses were performed using Stata (version 12.1, StataCorp, College Station, TX, USA). The reference standard for the diagnosis of PJP was > 1000 copies/mL of *P. jirovecii* DNA in sputum on real-time PCR, based on the cut-offs that distinguished colonisation from infection in two studies.^6,7^ Missing data for *P. jirovecii* copy number were imputed with other potentially predictive variables (age, sex, cough duration, dyspnoea, respiratory rate, oxygen saturation, chest radiograph likely/possible PJP, haemoglobin, WCC, CD4+ count, culture-positive tuberculosis, β-d-glucan concentrations, medical officer discharge diagnosis and use of antiretroviral therapy) using chained equations modelling and range matching of numeric values.

For the development of the clinical prediction rule, we conservatively estimated 10% of our cohort of 500 participants would have PJP. Four candidate predictor variables would then have more than 10 outcome events per variable, which is recommended for multivariable logistic regression analysis.^8^ The four candidate predictor variables were selected *a priori* for their ability to predict PJP and their accessibility in resource-poor settings: dyspnoea,^2^ haemoglobin,^9^ oxygen saturation,^10,11^ and chest radiographic features of PJP.^2,10,11^ Respiratory rate^12^ was explored as a replacement variable for oxygen saturation as pulse oximetry is not always available in resource-poor settings. We used univariable associations to assess the ability of the selected variables to predict the diagnosis of PJP. A backward stepwise approach proposed by Collet^13^ was used to select the most predictive variables in establishing a multivariable logistic regression model. Calibration of the model was visually assessed through a calibration plot and by the Hosmer-Lemeshow test (10 groups; 8 degrees of freedom). We used 1000 bootstrap re-samples for internal validation of our model. A clinical prediction rule with ready-to-use score chart was constructed utilising the method described by Sullivan et al.^14^ We used the clinical prediction rule to predict the probability of having PJP and compared it with the reference standard. We calculated the diagnostic accuracy for the range of possible scores from the clinical prediction rule and calculated the area under the curve (AUC) of the receiver operating characteristic (ROC) of the model to assess discrimination.

## Ethical consideration

Approval for the study was obtained from the University of Cape Town’s human research ethics committee. Eligible participants signed informed consent before enrolment into the study. Confused participants were enrolled and given the option to continue with participation once orientated; their data were removed from the study if consent was declined.

## Results

We enrolled 500 participants from November 2011 to October 2014. Sixteen participants just failed to meet criteria for WHO danger signs (e.g. temperature 39 ^°^C not > 39 ^°^C) or had missing data on danger signs, but were included in the analysis. The baseline characteristics of the participants are shown in Table 1. Most of the participants had more than one WHO danger sign. Note that the medical officer discharge diagnoses sum to more than 500 because of co-infections. Sputum PCR for *P. jirovecii* was > 1000 copies/mL in 15 of 29 participants who had sputum PCR done and had a medical officer discharge diagnosis of PJP.

**TABLE 1:** Baseline characteristics of 500 HIV-infected participants admitted with a cough of any duration and one or more World Health Organization danger signs.

Variable	Median (IQR)	*n* (%)
Age (years)	36 (30–42)	-
Sex (female)	-	327 (65)
BMI (kg/m^2^)	20.3 (18.0–24.4)^a^	-
CD4+ (cells/µL)	94 (35–216)^b^	-
On cotrimoxazole prophylaxis	-	121 (24)
Cough duration (days)	14 (7–21)^c^	-
Dyspnoea	*n* (%)^d^	372 (74)^d^
Oxygen saturation (%)	97 (94–98)^e^	-
On ART	-	174 (35)
Duration on ART (years)	2.3 (0.2–5.0)^f^	-
Previous tuberculosis	-	242 (48)
Chest X-ray possible/likely PJP	-	98 (20)^g^
Culture-positive tuberculosis	-	257 (51)
β-D-glucan (pg/mL)	67 (34–140)^h^	67 (34–140)
**WHO danger signs**		
Respiratory Rate > 30 breaths/min	-	315 (65)
Heart Rate > 120 beats/min	-	388 (78)
Temperature > 39 °C	-	87 (17)
Unable to walk unaided	-	259 (52)
**Haematological investigations**		
Haemoglobin (g/dL)	8.7 (7.9–11.2)^i^	-
WCC (x10^9^/L)	8.6 (5.6–12.9)^j^	-
**Medical officer discharge diagnosis**		
Tuberculosis	-	309 (62)
PJP	-	51 (10)
Bacterial pneumonia	-	258 (52)
Other	-	15 (3)

IQR, interquartile range; BMI, body mass index; ART, antiretroviral therapy; PJP, *Pneumocystis jirovecii* pneumonia.

a , 7 missing values;

b , 1 missing value;

c , 3 missing values;

d , 2 missing values;

e , 4 missing values;

f , 7 missing values;

g , 72 missing values;

h , 107 missing values and 7 values below the level of quantification of the assay;

i , 3 missing values;

j , 3 missing value.

Results of *P. jirovecii* in sputum on real-time PCR were available in 284 (57%) participants: 26 had > 1000 copies/mL, three had detectable *P. jirovecii* < 1000 copies/mL and the remainder had no detectable *P. jirovecii*. The yield of *P. jirovecii* > 1000 copies/mL was higher with induced versus spontaneous sputum (21/201 vs. 5/83; odds ratio 1.82; 95% CI 0.66–5.00). The final multivariable model of predictors of *P. jirovecii* copy number included the following five variables: chest radiograph showing possible/likely PJP, oxygen saturation, haemoglobin, CD4+ count and β-d-glucan (Table 2). The imputed data set was similar to the complete data. After imputation of missing data, there were 56 participants with > 1000 copies/mL of *P. jirovecii* in sputum on real-time PCR.

**TABLE 2:** The final multivariable model of predictors of *Pneumocystis jirovecii* copy number on sputum real-time polymerase chain reaction used for imputation.

Variable	β-coefficient (95% CI)	*p*
Chest X-ray possible/likely PJP	0.9026 (0.5274 to 1.2777)	< 0.001
Oxygen saturation	−0.0358 (−0.0636 to −0.0081)	0.012
Haemoglobin	0.1514 (0.0522 to 0.2506)	0.003
CD4+ count	−0.0015 (−0.0029 to −0.0001)	0.031
β-d-glucan	0.0074 (0.0056 to 0.0092)	< 0.001

CI, confidence interval; PJP, *Pneumocystis jirovecii* pneumonia.

Dyspnoea was not a significant predictor of PJP in the multivariable models and was dropped from the clinical prediction rules. We categorised haemoglobin by the median, rounded off from 8.7 to 9 g/dL. The final multivariable logistic regression models used to create the clinical prediction rules are shown in Table 3. There was an inverse correlation between oxygen saturation and respiratory rate (*r* = −0.288; *p* < 0.0001).

**TABLE 3:** The final multivariable logistic regression models used to create the clinical prediction rules.

Variable	Adjusted odds ratio (95% CI)	*p*
**Respiratory rate model**		
Chest X-ray possible/likely PJP	6.994 (3.769 to 12.979)	< 0.001
Haemoglobin ≥ 9 g/dL	2.518 (1.236 to 5.131)	0.011
Respiratory rate (per 10 increase)	2.199 (1.524 to 3.172)	< 0.001
**Oxygen saturation model**		
Chest X-ray possible/likely PJP	6.459 (3.455 to 12.074)	< 0.001
Haemoglobin ≥ 9 g/dL	2.071 (1.008 to 4.255)	0.048
Saturation < 94%	4.967 (2.643 to 9.334)	< 0.001

CI, confidence interval; PJP, *Pneumocystis jirovecii* pneumonia.

### Respiratory rate model

**FIGURE 1:**
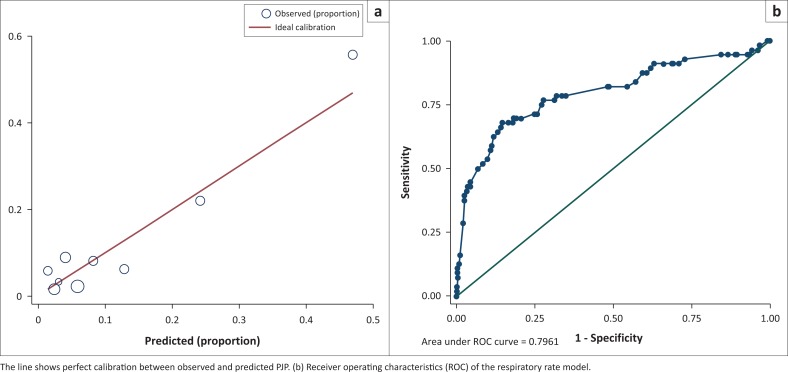
Respiratory rate multivariable logistic regression model to establish a clinical prediction rule for the diagnosis of *Pneumocystis jirovecii* pneumonia (PJP) among 500 seriously ill HIV-infected participants presenting with a cough of any duration and one or more World Health Organization danger signs. (a) Calibration plot for the assessment of variables included in the respiratory rate model. (b) Receiver operating characteristics (ROC) of the respiratory model.

The 3-variable logistic regression model had good calibration (Figure 1a) and followed the ideal calibration line, confirmed by the Hosmer-Lemeshow chi-squared statistic of 16.12 (*p* = 0.0644). The ROC AUC of the model was 0.7961 (95% CI 0.7216–0.8703) (Figure 1b). The equivalent AUC of the ROC in bootstrap validation was 0.7961 (95% CI 0.7216–0.8705). The optimism estimate was 0.0091347 (95% CI 0.006938–0.011331), indicating good stability of the model. The ROC AUC of the final optimism-corrected clinical prediction rule model was 0.7614 (95% CI 0.6833–0.8395).

### Oxygen saturation model

**FIGURE 2:**
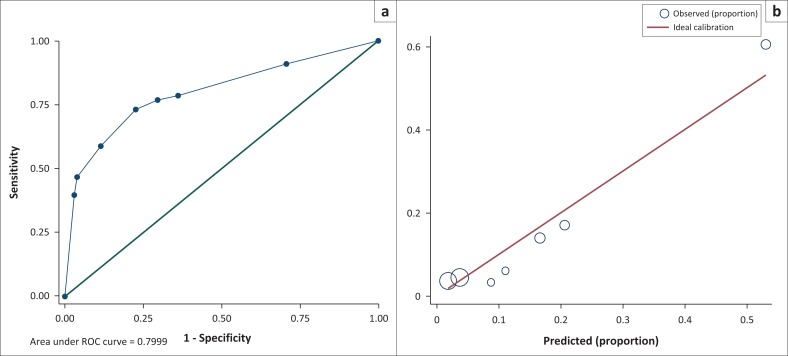
Oxygen saturation multivariable logistic regression model to establish a clinical prediction rule for the diagnosis of *Pneumocystis jirovecii* pneumonia (PJP) among 500 seriously ill HIV-infected participants presenting with a cough of any duration and one or more World Health Organization danger signs. (a) Calibration plot for the assessment of variables included in the oxygen saturation model. The line shows perfect calibration between observed and predicted PJP. (b) Receiver operating characteristics (ROC) of the oxygen saturation model.

We categorised oxygen saturation as the lowest quartile (< 94%). The 3-variable logistic regression model had good calibration (Figure 2a) and followed the ideal calibration line, confirmed by the Hosmer–Lemeshow chi-squared statistic of 5.96 (*p* = 0.5447). The AUC of the ROC of the model was 0.7999 (95% CI 0.7288–0.8715) (Figure 2). The equivalent AUC of the ROC in bootstrap validation was 0.7998 (95% CI 0.7283–0.8715). The optimism estimate was 0.005787 (95% CI 0.003612–0.007963), indicating good stability of the model. The ROC AUC of the final optimism-corrected clinical prediction rule model was 0.7969 (95% CI 0.7254–0.8684).

The ROC AUCs of the respiratory rate and oxygen saturation models were similar (*p* = 0.684; DeLong test, 1 degree of freedom). The clinical prediction rules derived from the respiratory rate and oxygen saturation multivariable logistic regression models are shown in Table 4 and the diagnostic accuracy assessment in Table 5.

**TABLE 4:** Clinical prediction rules derived from the respiratory rate and oxygen saturation multivariable logistic regression models for the diagnosis of *Pneumocystis jirovecii* pneumonia among 500 seriously ill HIV-infected participants presenting with a cough of any duration and one or more World Health Organization danger signs.

Variable	Points
**Respiratory rate model**	
Chest X-ray possible/likely PJP	2
Haemoglobin ≥ 9 g/dL	1
Respiratory rate 30–39	1
40–49	2
≥ 50	3
**Oxygen saturation model**	
Chest X-ray possible/likely PJP	3
Haemoglobin ≥ 9 g/dL	1
Saturation < 94%	2

PJP, *Pneumocystis jirovecii* pneumonia.

**TABLE 5:** Diagnostic accuracy assessment of the respiratory rate and oxygen saturation clinical prediction rules for the diagnosis of *Pneumocystis jirovecii* pneumonia among 500 seriously ill HIV-infected participants presenting with a cough of any duration and one or more World Health Organization danger signs.

Total score	Number with score	Probability of PJP	Sensitivity	Specificity	Likelihood ratio positive	Likelihood ratio negative
**Respiratory rate model**						
0	50	0	100.0%	0.0%	1.000	-
1	153	3.1%	94.6%	10.6%	1.059	0.506
2	154	6.5%	82.1%	43.5%	1.453	0.411
3	73	13.3%	69.6%	76.6%	2.973	0.396
4	45	25.2%	51.8%	90.8%	5.608	0.531
5	23	42.5%	26.8%	97.8%	11.893	0.749
6	2	61.9%	1.8%	99.8%	7.929	0.984
**Oxygen saturation model**						
0	135	0	100	0	1.000	-
1	161	3.9%	91.1%	29.3%	1.288	0.305
2	30	7.7%	78.6%	64.0%	2.180	0.335
3	90	14.8%	76.8%	70.5%	2.603	0.329
4	41	26.4%	58.9%	88.5%	5.130	0.464
5	8	42.6%	46.4%	96.2%	12.126	0.557
6	35	60.6%	39.3%	97.1%	13.418	0.626

PJP, *Pneumocystis jirovecii* pneumonia.

Note: Although it is possible to score 9 points in the respiratory rate clinical prediction rule, no participant had a score of higher than 6; therefore, the probability of PJP could not be calculated for higher scores.

## Discussion

We developed two clinical prediction rules for diagnosing PJP in HIV-infected inpatients with WHO danger signs and cough of any duration using variables readily available in resource-limited settings. The model based on respiratory rate had similar ROC AUC to the oxygen saturation model and could be implemented in resource-poor settings where pulse oximetry is not always available. Both clinical prediction rule models had acceptable to good discrimination as indicated by the ROC AUC of approximately 0.8. In both models, scores of zero were associated with zero probability of PJP, indicating value as rule out tests, and positive likelihood ratios exceeded 10 for high scores, indicating value as rule in tests. These clinical prediction rule models, if validated externally, could be incorporated into the WHO algorithm for seriously ill patients to guide decision-making about empiric therapy for PJP.

Dyspnoea was the only symptom consistently associated with PJP in a systematic review of studies conducted in LMICs,^2^ but dyspnoea was not a predictor of PJP in our cohort. A likely explanation for the poor predictive ability of dyspnoea in our cohort is that our patients had danger signs, which could reduce the ability of dyspnoea to discriminate PJP from other respiratory opportunistic diseases. We found that lower oxygen saturation and chest radiographic interpretation predicted PJP, in keeping with other studies.^2,10,11^ We also found that higher respiratory rate predicted PJP, but this has been an inconsistent finding in other studies.^11,12,15^

Our finding that serum (1→3)- β-d-glucan was associated with > 1000 copies/mL of *P. jirovecii* in sputum on multivariable analysis is in keeping with a systematic review, which reported the assay had a sensitivity of 92% and a specificity of 78% for the diagnosis of PJP in HIV-infected patients.^16^ However, this assay is expensive and is seldom available in resource-poor settings.

Our study has several limitations. First, a high proportion of our participants had missing values for our reference standard, sputum PCR for *P. jirovecii*. Sputum PCR values were missing at random because of a laboratory error resulting in the discarding of samples from a specific period when participants were enrolled, not because of any differences in the reasons for performing PCR. Other key variables with significant numbers of missing values were also missing at random: some chest radiographs went missing when one of the hospitals closed and serum (1→3)-β-d-glucan tests were only performed after the first few months of the study when additional funding became available. Because the data were missing at random, we were able to impute missing values, which has been shown to give better results than complete case analysis.^17^ Second, our findings were in inpatients with WHO danger signs in a population with a high prevalence of tuberculosis and may not be generalisable to other populations of HIV-infected patients. Third, when we conducted the study, the WHO algorithm required cough for more than two weeks,^18^ which we modified to any duration as tuberculosis commonly has an acute presentation in HIV-infected patients. The updated 2016 WHO algorithm^3^ requires only one of current cough, fever, night sweats or weight loss. However, it is very unlikely that patients with PJP would not have a cough and the updated WHO algorithm still includes the statement ‘consider treatment for *Pneumocystis* pneumonia’; therefore, we feel that our cohort is relevant to the 2016 WHO algorithm. Fourth, the quantitative real-time PCR cut-off for *P. jirovecii* that we used was based on two studies that developed cut-offs to distinguish between colonisation and infection,^6,7^ but there is as yet no standard cut-off.^19^ A meta-analysis found that real-time PCR has a sensitivity of 97% and a specificity of 93% for the diagnosis of PJP in HIV-infected patients,^19^ indicating that this test is a reasonable reference standard. Our yield of sputum PCR for *P. jirovecii* could have been higher if we had performed induced sputum in all participants.

Internal validation by bootstrapping resulted in a low optimism estimate, indicating good stability of the two models. However, our clinical prediction rules need to be externally validated as internal validation tends to be optimistic.

In conclusion, we developed two simple clinical prediction rule models to facilitate the diagnosis of PJP in HIV-infected inpatients with WHO danger signs in resource-limited settings. There is a need for external validation, ideally in a different geographic setting, before these models can be considered for incorporation into a revised WHO seriously ill algorithm or in HIV-infected patients without WHO danger signs.
